# TP53 Functional-Domain-Specific Mutations Define Distinct Clinical Outcomes in EGFR-Mutant Non-Small Cell Lung Cancer Treated with EGFR Tyrosine Kinase Inhibitors

**DOI:** 10.3390/jcm15041552

**Published:** 2026-02-15

**Authors:** Keigo Kobayashi

**Affiliations:** 1 Division of Medical Oncology, National Cancer Centre Singapore, 30 Hospital Blvd, Singapore 168583, Singapore; keigokbys@gmail.com; 2 Department of Internal Medicine, Japan Green Vietnam Clinic, Hoang Minh Thao Street, Xuan Dinh Ward, Hanoi 11910, Vietnam

**Keywords:** EGFR-mutant non-small cell lung cancer, TP53 functional domain mutation, DNA-binding domain, EGFR tyrosine kinase inhibitors, prognostic stratification, clinical decision making

## Abstract

**Background:** In advanced non-small cell lung cancer (NSCLC) with sensitizing EGFR mutations, EGFR tyrosine kinase inhibitors (EGFR-TKIs) improve progression-free survival (PFS). However, clinical outcomes vary according to EGFR mutation subtype and TP53 co-mutations. Most prior studies have evaluated TP53 status as binary, and the clinical relevance of domain-specific TP53 alterations remains insufficiently defined. **Methods:** We retrospectively analyzed patients with advanced NSCLC harboring sensitizing EGFR mutations who received first-line EGFR-TKI therapy at the National Cancer Centre Singapore between 22 November 2007, and 17 February 2022. EGFR mutations were classified as common (exon 19 deletion or L858R) or uncommon (all others). TP53 alterations were categorized into three groups: (i) DNA-binding domain (DBD)-involved mutations, including DBD-only mutations and those with additional oligomerization domain (OD) involvement; (ii) other TP53 mutations not involving the DBD or OD; and (iii) TP53 wild type (TP53-WT). The primary endpoint was PFS. Survival analyses were performed using the Kaplan–Meier method and Cox proportional hazards models. **Results:** TP53 alterations were identified in approximately half of the cohort and were predominantly concentrated within the DBD. In the overall cohort, patients treated with third-generation EGFR-TKIs had longer PFS than those treated with first- or second-generation EGFR-TKIs, with this difference being more pronounced among patients with TP53-mutant tumors; no clear PFS difference by TKI generation was observed in the TP53-WT subgroup. Patients with common EGFR mutations experienced significantly longer PFS than those with uncommon mutations, particularly in the presence of TP53 co-mutations. Across multiple analyses, TP53 DBD-involved mutations were associated with shorter PFS compared with other TP53 mutations and TP53-WT, especially in patients treated with first- or second-generation EGFR-TKIs and in those with common EGFR mutations. **Conclusions:** In EGFR-mutant NSCLC treated with EGFR-TKIs, TP53 functional domain involvement provides prognostic information beyond TP53 mutation status alone. TP53 DBD-involved alterations define a high-risk subgroup with inferior PFS, particularly in treatment settings using first- or second-generation EGFR-TKIs. Incorporation of TP53 domain-based classification, together with EGFR mutation subtype, may improve risk stratification and help guide treatment planning in EGFR-mutant NSCLC.

## 1. Introduction

For patients with advanced non-small cell lung cancer (NSCLC) harboring sensitizing EGFR mutations (e.g., exon 19 deletions and L858R), multiple phase III trials have established that EGFR tyrosine kinase inhibitors (EGFR-TKIs) significantly prolong progression-free survival (PFS) compared with platinum-based chemotherapy [1,2,3]. In recent years, osimertinib, a third-generation EGFR-TKI, has demonstrated superior PFS and overall survival (OS) over first- and second-generation EGFR-TKIs in the first-line setting and has become the standard of care for EGFR-mutant NSCLC [4].

EGFR mutations are broadly categorized into common mutations (exon 19 deletions and L858R) and uncommon mutations. Uncommon mutations are molecularly heterogeneous, with a wide range of sensitivity to EGFR-TKIs, and certain uncommon subtypes are associated with poor clinical outcomes [5,6]. In addition, TP53 co-mutations are frequently observed in EGFR-mutant NSCLC and have been consistently identified as an adverse prognostic factor after EGFR-TKI therapy in multiple retrospective studies and meta-analyses [7,8,9].

TP53 mutations are biologically diverse, and tumor behavior varies substantially depending on the mutation type and the affected functional domain. In particular, missense mutations within the DNA-binding domain (DBD), which account for the majority of TP53 alterations, not only abrogate the tumor-suppressive functions of wild-type p53 but may also confer oncogenic gain-of-function (GOF) properties [10,11]. Furthermore, the oligomerization domain (OD) is essential for p53 tetramerization. Although isolated OD mutations are rare, OD involvement co-occurring with DBD involvement may exert a dominant-negative effect on residual wild-type p53 activity and thereby amplify or modify DBD-related dysfunction [12].

Despite these biological insights, most clinical studies in EGFR-mutant NSCLC have evaluated TP53 status simply as “mutant” versus “wild-type,” and clinical investigations focusing on domain-specific effects—particularly mutations involving the DBD (DBD-involved mutations)—remain limited [13]. Therefore, we aimed to examine in detail the impact of TP53 mutation status and its functional subtypes on PFS after EGFR-TKI therapy in EGFR-mutant NSCLC.

## 2. Materials and Methods

### 2.1. Patient Population

Patients with advanced non-small cell lung cancer (NSCLC) harboring sensitizing EGFR mutations were screened at the National Cancer Centre Singapore from 22 November 2007 onward. Diagnosis and treatment were performed in routine clinical practice, and clinical outcomes were tracked from the initiation of EGFR-TKI therapy.

Among the screened population, patients who received first-line EGFR-TKI therapy were identified from 2012. For the present analysis, we focused on patients who received palliative first-line EGFR-TKI treatment and had available comprehensive EGFR and TP53 genomic profiling results. First-line EGFR-TKI therapy was defined as the first systemic treatment for recurrent or advanced disease. Accordingly, EGFR T790M or C797S mutations detected at baseline were not considered de novo if patients had prior EGFR-TKI exposure. This resulted in a final analytic cohort of 208 patients who initiated EGFR-TKI therapy between September 2012 and January 2022.

**Figure 1:**
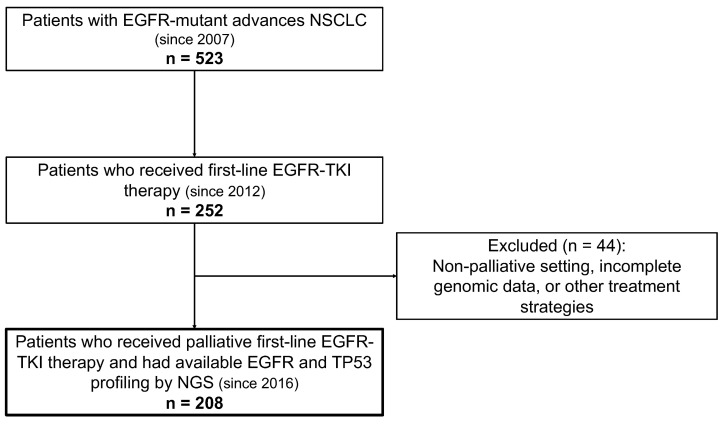
Patient selection flowchart. Flow diagram of patient selection and derivation of the analytic cohort. The diagram illustrates the screening population, treatment-based selection, and the final NGS-analyzed cohort.

The patient-selection process and derivation of the analytic cohort are summarized in Figure 1.

### 2.2. Genomic Analysis

Comprehensive genomic profiling of EGFR and TP53 was performed using a single institutionally validated targeted next-generation sequencing (NGS) panel (Singapore Solid Tumour Panel; SSTP) in all patients included in the analytic cohort.

NGS testing was implemented from 2016 onward, and only patients with available NGS-based genomic profiling were included in the present analysis. Therefore, no mixture of PCR- and NGS-based platforms was present within the analyzed cohort, minimizing potential temporal or technical heterogeneity in TP53 mutation detection.

The assay provided high sequencing depth, with near-complete panel coverage at ≥250× and substantial coverage at ≥500–1000×, enabling reliable detection of single-nucleotide variants and small insertions/deletions across the full coding regions of TP53, including the DNA-binding domain.

EGFR mutations were classified as common or uncommon. Common EGFR mutations were strictly defined as isolated exon 19 deletions or isolated L858R substitutions. All other EGFR alterations were classified as uncommon mutations, including uncommon point mutations, exon 20 insertions or duplications, and compound mutations in which exon 19 deletions or L858R co-occurred with additional EGFR variants (e.g., T790M or C797S).

For TP53, mutation status was first determined as mutant or wild type. TP53-mutant cases were then categorized into three groups based on the functional domains involved:

(1) DBD-involved TP53 mutations, defined as mutations involving the DNA-binding domain (DBD), including DBD-only mutations and complex mutations with additional oligomerization domain (OD) involvement;
(2) Other TP53 mutations (DBD/OD-non-involved), defined as mutations involving neither the DBD nor the OD (e.g., transactivation domain mutations, frameshift mutations, splice-site mutations);
(3) TP53 wild type, defined as no detectable TP53 mutations.

### 2.3. Study Endpoints

The primary endpoint was PFS. Because follow-up was relatively short and the number of OS events was judged to be insufficiently mature, OS was not included as a primary endpoint and was evaluated in an exploratory manner.

EGFR-TKI efficacy is often evaluated more directly using PFS, and PFS has been adopted as the primary endpoint in pivotal phase III trials of EGFR-mutant NSCLC [2,4]. In contrast, OS analyses require longer follow-up and can be strongly influenced by subsequent therapies and crossover, and thus OS was not selected as the primary endpoint in this study.

### 2.4. Statistical Analysis

PFS was defined as the time from initiation of EGFR-TKI therapy to disease progression or death from any cause. Patients without progression at the time of the last follow-up were censored.

PFS curves were estimated using the Kaplan–Meier method, and differences between groups were assessed using the log-rank test. Hazard ratios (HRs) and 95% confidence intervals (CIs) were estimated using Cox proportional hazards models. To evaluate whether TP53 functional subtypes were independently associated with progression-free survival, multivariable Cox proportional hazards regression models were constructed. Covariates included age, sex, EGFR mutation subtype (common vs. uncommon), first-line EGFR-TKI generation (first/second generation vs. third generation), and TNM stage (stage IVB vs. IVA), which were selected a priori based on clinical relevance. Because only two patients had stage III disease, these cases were excluded from the multivariable analysis to ensure model stability.

Hazard ratios (HRs) and 95% confidence intervals (CIs) were estimated. All statistical tests were two-sided, and *p* values < 0.05 were considered statistically significant.

OS was analyzed exploratorily and is presented in the Supplementary Materials because of limited follow-up and an immature number of events.

### 2.5. Ethics Statement

This retrospective observational study used fully anonymized clinical data obtained from the National Cancer Centre Singapore. According to local institutional policies and national ethical guidelines in Singapore, ethical review and Institutional Review Board (IRB) approval were waived.

Informed consent was also waived due to the retrospective nature of the study and the use of fully anonymized data.

## 3. Results

### 3.1. Patient Characteristics

Baseline characteristics of the overall population and those stratified by TP53 functional subgroups are shown in Table 1a and Table 1b, respectively. Overall, the cohort’s clinical features—including age, sex, smoking history, and histology—were broadly consistent with those reported in prior EGFR-TKI studies in advanced EGFR-mutant NSCLC. Common EGFR mutations, defined as exon 19 deletions and L858R, accounted for the majority of EGFR alterations.

### 3.2. Distribution of TP53 Mutations

**Figure 2:**
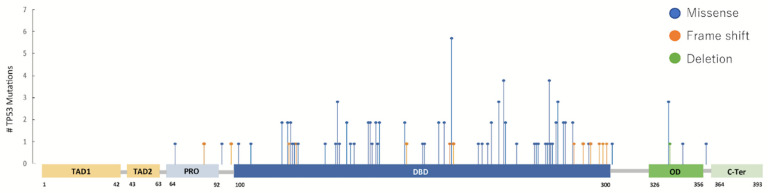
Distribution of TP53 mutations across the TP53 gene. Lollipop plot illustrating the distribution of TP53 mutations detected in the study cohort. Each lollipop represents an individual mutation, plotted according to its amino acid position along the TP53 protein. Mutations are predominantly clustered within the DNA-binding domain (DBD), whereas mutations in the transactivation domain and other regions are less frequent. Truncating mutations, including frameshift and splice-site variants, were rare in this cohort. TAD, transactivation domain; PRO, proline-rich domain; DBD, DNA-binding domain; OD, oligomerization domain; C-ter, C-terminal domain.

To visualize the distribution of TP53 mutations, a lollipop plot was generated (Figure 2). Although TP53 mutations were distributed across the gene, they were predominantly concentrated within the DBD, indicating that the DBD represented the major mutational hotspot in this cohort. In contrast, mutations in the transactivation domain and other regions were relatively infrequent, and truncating alterations such as frameshift or splice-site mutations were rare. Collectively, these findings indicate that TP53 alterations in this cohort were enriched in the functionally critical DBD.

### 3.3. PFS by First-Line EGFR-TKI Generation and TP53 Status (Figure 3)

In the overall cohort, patients who received third-generation EGFR-TKIs as first-line therapy experienced longer PFS than those treated with first- or second-generation EGFR-TKIs. When the analysis was restricted to TP53-mutant patients, this difference became more pronounced, with clear PFS prolongation observed in patients treated with third-generation EGFR-TKIs. In contrast, among TP53-WT patients, no clear PFS difference was observed between EGFR-TKI generations.

**Figure 3:**
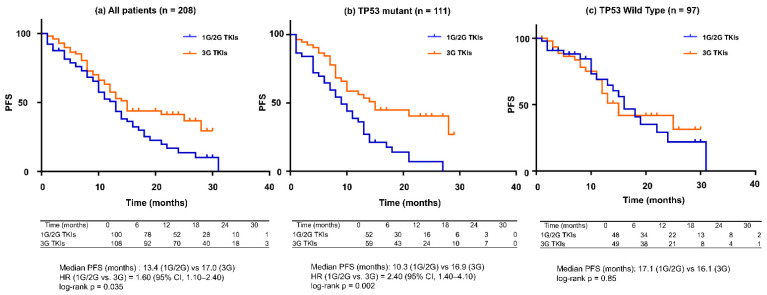
Progression-free survival (PFS) compared between 1G/2G TKIs and 3G TKIs according to TP53 mutation status. Kaplan–Meier curves for PFS are shown for (**a**) the overall cohort, (**b**) patients with TP53-mutant tumors, and (**c**) patients with TP53 wild-type tumors. Blue lines represent patients treated with first- or second-generation EGFR-TKIs (1G/2G TKIs), and orange lines represent those treated with third-generation EGFR-TKIs (3G TKIs). Numbers at risk are shown below each panel. (**a**) All patients (*n* = 208): Patients treated with 3G TKIs experienced significantly longer PFS than those treated with 1G/2G TKIs (median PFS, 17.0 vs. 13.4 months; hazard ratio [HR] for 1G/2G vs. 3G, 1.60; 95% CI, 1.10–2.40; log-rank *p* = 0.035). (**b**) TP53-mutant group (*n* = 111): The PFS benefit of 3G TKIs was more pronounced among patients with TP53-mutant tumors (median PFS, 16.9 vs. 10.3 months; HR, 2.40; 95% CI, 1.40–4.10; log-rank *p* = 0.002). (**c**) TP53 wild-type group (*n* = 97): No significant difference in PFS was observed between patients treated with 1G/2G TKIs and those treated with 3G TKIs (median PFS, 17.1 vs. 16.1 months; log-rank *p* = 0.85).

### 3.4. PFS by EGFR Mutation Subtype and TP53 Status (Figure 4)

In analyses stratified by EGFR mutation subtype, patients with common EGFR mutations exhibited significantly longer PFS than those with uncommon EGFR mutations. This trend was consistently observed in the TP53-mutant subgroup, in which patients harboring common EGFR mutations showed relatively favorable PFS, whereas those with uncommon EGFR mutations experienced markedly shorter PFS.

**Figure 4:**
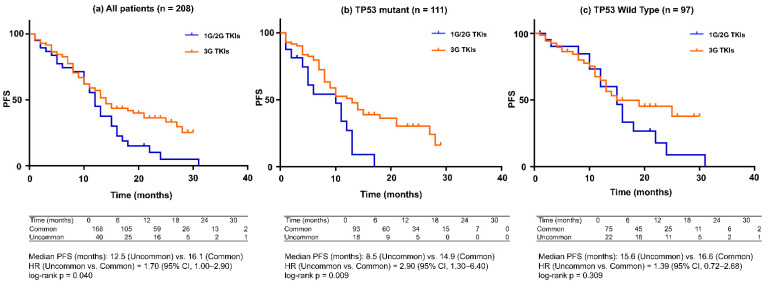
Progression-free survival (PFS) according to EGFR mutation type (Common vs. Uncommon) stratified by TP53 mutation status. Kaplan–Meier curves for PFS are shown for (**a**) the overall cohort, (**b**) patients with TP53-mutant tumors, and (**c**) patients with TP53 wild-type tumors. Blue lines represent patients with common EGFR mutations (exon 19 deletion or L858R), and orange lines represent those with uncommon EGFR mutations. (**a**) All patients (*n* = 208): Patients with common EGFR mutations had significantly longer PFS than those with uncommon EGFR mutations (median PFS, 16.1 vs. 12.5 months; hazard ratio [HR] for uncommon vs. common, 1.70; 95% CI, 1.00–2.90; log-rank *p* = 0.040). (**b**) TP53-mutant group (*n* = 111): Among patients with TP53-mutant tumors, uncommon EGFR mutations were associated with a markedly shorter PFS compared with common mutations (median PFS, 8.5 vs. 14.9 months; HR, 2.90; 95% CI, 1.30–6.40; log-rank *p* = 0.009). (**c**) TP53 wild-type group (*n* = 97): No significant difference in PFS was observed between common and uncommon EGFR mutations in the TP53 wild-type subgroup (median PFS, 16.6 vs. 15.6 months; HR, 1.39; 95% CI, 0.72–2.68; log-rank *p* = 0.309).

In contrast, among patients with TP53 wild-type tumors, no significant difference in PFS was observed between common and uncommon EGFR mutation subtypes. Notably, the uncommon EGFR mutation group was highly heterogeneous, comprising various compound mutations, exon 20 insertions, and rare point mutations (Supplementary Materials Table S1). This heterogeneity may have limited the ability to detect prognostic differences within TP53-defined subgroups.

When analyses were restricted to patients with EGFR mutations treated with third-generation EGFR-TKIs, no significant differences in PFS were observed among TP53 functional subtypes by either log-rank testing or Cox proportional hazards modeling (Figure 4).

### 3.5. PFS by TP53 Functional Subtype (Figure 5)

In analyses stratified by TP53 functional subtype, patients with DBD-involved TP53 mutations had the shortest PFS compared with those with other TP53 mutations (DBD/OD-non-involved) and those with TP53 wild type. This trend was consistently observed in analyses restricted to patients treated with first- or second-generation EGFR-TKIs and in analyses restricted to patients harboring common EGFR mutations.

**Figure 5:**
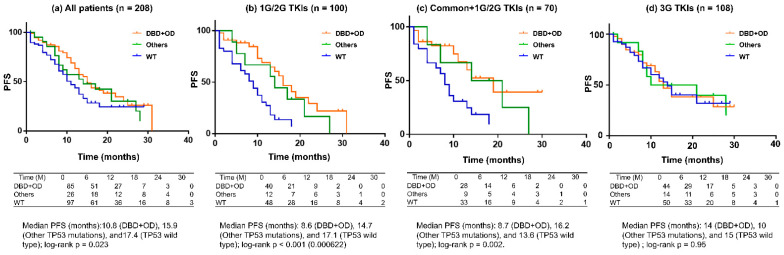
Kaplan–Meier curves of progression-free survival (PFS) according to *TP53* mutation subtypes classified by functional domains. PFS was compared among three groups: patients with TP53 mutations involving the DNA-binding domain (DBD), including mutations with or without additional oligomerization domain (OD) involvement (DBD-involved; blue lines); patients with other TP53 mutations not involving the DBD or OD (Other mutations; green lines); and patients with TP53 wild-type tumors (Wild type; orange lines). (**a**) All patients (*n* = 208): Patients with DBD-involved TP53 mutations exhibited significantly shorter PFS compared with those harboring other TP53 mutations or TP53 wild-type tumors (log-rank *p* < 0.05). (**b**) Patients treated with first- or second-generation EGFR-TKIs (*n* = 100): The adverse prognostic impact of DBD-involved TP53 mutations was more pronounced in this subgroup, with significantly shorter PFS compared with the other two TP53 subgroups (log-rank *p* < 0.01). (**c**) Patients with common EGFR mutations treated with first- or second-generation EGFR-TKIs (*n* = 70): DBD-involved TP53 mutations remained a significant poor prognostic factor in this clinically favorable EGFR subgroup (log-rank *p* < 0.05). (**d**) Patients treated with third-generation EGFR-TKIs (*n* = 108): No significant differences in PFS were observed among TP53 functional subgroups (log-rank *p* = n.s.). Hazard ratios estimated using Cox proportional hazards models corresponding to each panel are provided in Supplementary Materials Table S2.

To quantify the effect size among the three TP53 subgroups, Cox proportional hazards models were applied. Compared with TP53 wild-type patients, those with DBD+OD mutations had significantly shorter PFS (HR 1.95, 95% CI 1.23–3.09), whereas other TP53 mutations were not significantly associated with PFS (HR 1.27, 95% CI 0.78–2.06). When TP53 status was modeled as an ordinal variable, a significant trend toward worse PFS was observed (HR per step 1.28, 95% CI 1.03–1.59; *p* = 0.023). The results of the multivariable Cox analysis are summarized in Table 2.

In addition, to complement the Kaplan–Meier analyses shown in Figure 5, Cox proportional hazards models were applied separately for each corresponding subgroup analysis (overall cohort, first-/second-generation EGFR-TKIs, common EGFR mutations treated with first-/second-generation EGFR-TKIs, and third-generation EGFR-TKIs).

Consistent with the Kaplan–Meier results, TP53 DBD-involved mutations were associated with a significantly higher risk of progression in the overall cohort and in patients treated with first- or second-generation EGFR-TKIs, whereas no significant differences were observed among TP53 subgroups in patients treated with third-generation EGFR-TKIs. These results are summarized in Supplementary Materials Table S2.

Overall survival (OS) was analyzed in an exploratory manner and is presented in Supplementary Figure S1. No statistically significant difference in OS was observed according to EGFR-TKI generation (first/second generation vs. third generation; log-rank *p* = 0.266).

## 4. Discussion

### 4.1. Patient Characteristics and TP53 Mutation Distribution

The clinical profile of our cohort was broadly consistent with that of previously reported populations of advanced EGFR-mutant NSCLC, including distributions of age, sex, smoking history, and histology [2,14]. Most patients had adenocarcinoma and were never-smokers or light smokers, aligning with the typical phenotype of EGFR-mutant NSCLC [15]. Common EGFR mutations (exon 19 deletions and L858R) accounted for the majority of cases, consistent with prior epidemiologic reports [4], while a meaningful fraction of uncommon mutation cases supported the rationale for EGFR subtype-stratified analyses.

TP53 mutations were detected in approximately half of patients, which falls within the range reported for TP53 co-mutations in EGFR-mutant lung cancer [8,13]. Notably, TP53 mutations were enriched within the DBD rather than being randomly distributed, underscoring the biological relevance of domain-specific stratification and supporting our approach of classifying TP53 mutations by functional domain instead of treating all TP53 alterations as a single entity [10,11].

Our cohort included patients treated with both first-/second-generation and third-generation EGFR-TKIs. Although this heterogeneity could introduce analytic complexity, it also reflects real-world practice and may enhance external validity. Importantly, the observed PFS differences by TP53 status and EGFR subtype remained consistent in analyses restricted by EGFR-TKI generation, suggesting that these findings are not readily explained by baseline clinical heterogeneity alone but may reflect underlying biological differences.

Furthermore, major imbalances in basic clinical factors such as age and sex across TP53 categories were not observed, supporting the interpretation that the observed PFS differences are more likely attributable to molecular tumor characteristics. Overall, our cohort was representative of EGFR-mutant NSCLC and displayed sufficient diversity in TP53 alterations to assess the clinical relevance of TP53 functional heterogeneity.

### 4.2. Functional Heterogeneity of TP53 Mutations and the Rationale for Our Classification

TP53 mutations vary substantially in biological behavior depending on the affected functional domains [10,11]. Nonetheless, many clinical studies in EGFR-mutant NSCLC have categorized TP53 merely as mutated versus wild-type [7,8]. In this study, we sought to address this limitation by stratifying TP53-mutant cases according to functional domain involvement and examining the relationship between these subtypes and outcomes after EGFR-TKI therapy.

The DBD is the core region mediating p53-dependent transcriptional regulation, and TP53 mutations frequently cluster within this domain [10]. DBD missense mutations can lead not only to loss of tumor-suppressive activity but also to GOF properties that may promote tumor progression, therapeutic resistance, and genomic instability [10,11]. Associations between TP53 alterations and resistance to EGFR-TKIs have also been primarily discussed in the context of DBD missense mutations [7,8].

In contrast, TP53 mutations occurring outside the DBD—such as those in the transactivation domain or proline-rich region—are often considered biologically distinct. These alterations commonly result in reduced transcriptional activity or quantitative loss-of-function, and consistent GOF-like behavior or a clear link to therapeutic resistance comparable to DBD missense mutations has not been sufficiently demonstrated [10,16]. Similarly, truncating mutations (e.g., frameshift or splice-site mutations) often lead to complete functional loss, but they do not necessarily confer the same clinical aggressiveness as DBD missense mutations, and reported associations with outcomes after EGFR-TKI therapy have been inconsistent [8].

The OD is required for p53 tetramerization. While isolated OD mutations are rare, OD involvement co-occurring with DBD involvement may suppress residual wild-type p53 function via dominant-negative effects and thereby exacerbate DBD-related dysfunction [12]. Accordingly, we classified TP53 alterations into three categories: (i) DBD-involved mutations (including DBD-only and DBD with additional OD involvement), (ii) other TP53 mutations that involve neither DBD nor OD (DBD/OD-non-involved), and (iii) TP53 wild type. This framework was designed to reflect functional heterogeneity while remaining clinically interpretable under sample-size constraints.

Using this classification, we observed that DBD-involved TP53 mutations were consistently associated with the shortest PFS compared with other TP53 mutations (DBD/OD-non-involved) and TP53 wild type. This finding suggests that, in the setting of EGFR-TKI therapy, the prognostic significance of TP53 may depend not only on the presence of mutation but also on which functional domain is affected.

Because the number of cases with complex DBD-involved mutations with additional OD involvement was limited, we were unable to evaluate them as an independent subgroup and therefore included them within the DBD-involved category. Nonetheless, it remains possible that such complex DBD-involved alterations contributed to the poor PFS observed in this group, warranting validation in larger cohorts and functional studies.

### 4.3. Clinical Implications in the Context of First-Line EGFR-TKI Selection

Our results suggest that the effectiveness of first-line EGFR-TKI therapy in EGFR-mutant NSCLC may differ substantially according to TP53 mutation status and functional subtype. Notably, in Figure 3, the relative advantage of third-generation EGFR-TKIs over first-/second-generation EGFR-TKIs appeared more pronounced among TP53-mutant patients, whereas such a difference was not clearly observed in TP53 wild-type patients. This pattern highlights a clinically important possibility: the benefit of third-generation EGFR-TKIs may be maximized precisely in patients with TP53 alterations who are otherwise considered to have poorer prognoses.

In addition, Figure 4 indicates that the combination of uncommon EGFR mutations and TP53 mutations identifies a subgroup with particularly unfavorable outcomes, suggesting that the prognostic impact of TP53 may be amplified in the context of EGFR variants for which effective targeted options are limited or heterogeneously active.

Most importantly, Figure 5 demonstrates that TP53 functional subtype matters: DBD-involved mutations were consistently associated with the shortest PFS, and this trend persisted even when restricting analyses to first-/second-generation EGFR-TKI–treated cases and to patients with common EGFR mutations. These findings support the existence of a biologically aggressive subgroup that cannot be fully explained by EGFR subtype or treatment generation alone.

From a practical clinical standpoint, these results suggest that EGFR-mutant NSCLC with TP53 DBD-involved alterations may represent a high-risk population in which reliance on EGFR-TKI monotherapy alone may be suboptimal. Such patients may benefit from closer monitoring, earlier consideration of therapeutic switching, combination strategies, or enrollment in clinical trials. Overall, our findings support moving beyond a binary evaluation of TP53 status and instead incorporating TP53 functional domain involvement—together with EGFR mutation subtype—into risk stratification and treatment planning in EGFR-mutant NSCLC.

Importantly, the adverse prognostic impact of TP53 DBD-involved mutations remained significant after adjustment for age, sex, EGFR mutation subtype, and EGFR-TKI generation, indicating that TP53 functional domain involvement represents an independent prognostic factor rather than a surrogate for other clinical variables.

Notably, this TP53 subtype-specific prognostic effect was not observed in patients treated with third-generation EGFR-TKIs. Several explanations may account for this finding. Third-generation EGFR-TKIs, such as osimertinib, provide more potent and selective inhibition of EGFR signaling than first- and second-generation agents, resulting in more uniform and durable disease control in patients with EGFR common mutations [4]. This strong treatment effect may attenuate the prognostic influence of co-occurring tumor suppressor alterations, including TP53, which have been consistently associated with inferior outcomes in less effective treatment contexts [8].

Previous studies have reported that TP53 mutations are strongly associated with poorer outcomes in patients treated with first- or second-generation EGFR-TKIs [8], whereas their prognostic impact appears less pronounced in patients receiving osimertinib, particularly in the first-line setting [17]. These observations support the concept that the clinical relevance of TP53 alterations is treatment-context dependent rather than universal [18]. In addition, the relatively long PFS and limited number of progression events in the third-generation EGFR-TKI cohort may have reduced the statistical power to detect modest differences among TP53 subgroups. Therefore, the absence of statistical significance in this setting should not be interpreted as evidence that TP53 status is biologically irrelevant, but rather that its prognostic effect may be masked by the strong efficacy of third-generation EGFR-TKIs.

The exploratory OS analysis should be interpreted with caution. The third-generation EGFR-TKI group had a shorter median follow-up and fewer death events, indicating that OS data in this group remain immature. In addition, OS may be substantially influenced by subsequent therapies after disease progression, which limits its interpretability in this retrospective setting.

## 5. Limitations

This study has several limitations. First, its retrospective design introduces potential selection bias. Second, OS was not suitable as a primary endpoint because of limited follow-up duration and an immature number of events. Third, the limited number of cases in certain TP53 subgroups—particularly rare complex DBD-involved alterations—precluded independent subgroup analyses. In addition, the relatively small sample size and limited number of progression events in the third-generation EGFR-TKI–treated subgroup may have reduced the statistical power to detect modest prognostic differences among TP53 functional subtypes in this treatment context. Finally, as an observational clinical study, these findings lack direct experimental validation of the functional consequences of each TP53 subtype.

## 6. Conclusions

In conclusion, our study demonstrates that TP53 mutations in EGFR-mutant non-small cell lung cancer are clinically heterogeneous, and that mutations involving the DNA-binding domain (DBD-involved mutations) identify a subgroup of patients with particularly unfavorable progression-free survival after EGFR-TKI therapy.

Beyond the presence of TP53 mutation itself, functional domain-based classification provides improved prognostic stratification, with DBD-involved mutations associated with inferior outcomes in multiple clinically relevant settings, particularly among patients treated with first- or second-generation EGFR-TKIs and those harboring common EGFR mutations.

Moreover, the relative benefit of third-generation EGFR-TKIs appeared more evident in patients with TP53-mutant tumors, whereas no clear advantage by TKI generation was observed in TP53 wild-type disease, suggesting that TP53 status may be useful for contextualizing treatment response patterns rather than serving as a standalone determinant of therapy selection.

Together, these findings support the potential clinical utility of incorporating TP53 functional domain information at diagnosis to refine risk stratification and to better understand treatment outcomes in EGFR-mutant NSCLC.

## Figures and Tables

**Table 1:** Baseline patient characteristics. (**a**). Baseline characteristics of the overall study population. (**b**). Baseline characteristics stratified by TP53 functional subgroups.

(a)				
**Variable**	**Overall**			
Sex, *n* (%)				
Female	125 (60.0)			
Male	83 (40.0)			
Age at start of first-line therapy, years—Mean ± SD	66.8 ± 10.6			
Smoking status, *n* (%)				
Never smoker	162 (77.9)			
Former smoker	30 (14.4)			
Current smoker	16 (7.7)			
Histology, *n* (%)				
Adenocarcinoma	201 (96.6)			
Others	7 (3.4)			
Stage (8th edition), *n* (%)				
IIIA	1 (0.5)			
IIIB	1 (0.5)			
IVA	69 (33.2)			
IVB	137 (65.9)			
EGFR mutation, *n* (%)				
Exon 19 deletion	103 (49.5)			
L858R	65 (31.3)			
Others	40 (19.2)			
Type of first-line EGFR-TKI, *n* (%)				
Osimertinib	107 (51.4)			
Afatinib	35 (16.8)			
Gefitinib	26 (12.5)			
Erlotinib	22 (10.6)			
Dacomitinib	17 (8.2)			
Nazartinib	1 (0.5)			
Type of first-line EGFR-TKI, *n* (%)				
1st/2nd generation TKI	100 (48.1)			
3rd generation TKI	108 (51.9)			
Type of first-line EGFR-TKI, *n* (%)—Therapy response, *n* (%)	*n* = 181			
Complete response (CR)	0 (0.0)			
Partial response (PR)	135 (74.6)			
Stable disease (SD)	10 (5.5)			
Progressive disease (PD)	26 (14.4)			
Others	10 (5.5)			
TP53 status, *n* (%)				
Wild type	97 (46.6)			
Mutated	111 (53.4)			
TP53 mutation subtype (mutated cases)	*n* = 111			
Missense mutation	86 (77.5)			
Frameshift mutation	15 (13.5)			
Splicing mutation	5 (4.5)			
Deletion mutation	1 (0.9)			
Deletion/insertion mutation	1 (0.9)			
Other types	3 (2.7)			
(b)				
**Variable**	**TP53 DBD-Involved**	**TP53 Other**	**TP53 WT**	***p* Value**
Age at diagnosis, years—Median (range)	67 (50–89)	67 (40–87)	67 (32–94)	0.328
Sex, *n* (%)				
Female	54 (68.4)	16 (69.6)	55 (51.9)	
Male	25 (31.6)	7 (30.4)	51 (48.1)	0.0477
Smoking status, *n* (%)				
Never	72 (91.1)	14 (60.9)	76 (71.7)	
Former	6 (7.6)	6 (26.1)	18 (17.0)	
Current	1 (1.3)	3 (13.0)	12 (11.3)	0.0042
EGFR mutation subtype, *n* (%)				
Common	62 (78.5)	19 (82.6)	81 (76.4)	
Uncommon	17 (21.5)	4 (17.4)	25 (23.6)	0.800
First-line EGFR-TKI, *n* (%)				
1st generation	23 (29.1)	4 (17.4)	21 (19.8)	
2nd generation	25 (31.6)	3 (13.0)	24 (22.6)	
3rd generation	31 (39.2)	16 (69.6)	61 (57.5)	0.050

Table 1a summarizes the baseline demographic, clinical, and molecular characteristics of the overall study population. Table 1b presents baseline characteristics stratified by TP53 functional subgroups. EGFR common mutations were defined as isolated exon 19 deletions or isolated L858R substitutions; all other EGFR alterations were classified as uncommon mutations. Detailed patterns of uncommon EGFR mutations are provided in Supplementary Materials Table S1. Values are presented as mean ± standard deviation or number (%).

**Table 2:** Multivariable Cox proportional hazards analysis for progression-free survival (PFS).

Variable	Hazard Ratio	95% CI	*p* Value
TP53 functional subtype			
Others vs. WT	1.41	0.78–2.54	0.259
DBD-involved vs. WT	1.85	1.17–2.91	0.0085
Age (per 1-year increase)	1.00	0.98–1.02	0.772
Male vs. Female	1.26	0.84–1.90	0.269
EGFR uncommon vs. common	1.47	0.90–2.41	0.123
1G/2G vs. 3G EGFR-TKI	1.56	1.01–2.40	0.046
Stage IVB vs. IVA	1.55	0.98–2.44	0.06

Hazard ratios were estimated using a multivariable Cox proportional hazards model adjusting for age, sex, EGFR mutation subtype, EGFR-TKI generation, and TNM stage (IVA vs. IVB). Reference categories: WT, Female, EGFR common mutation, 3G EGFR-TKI, Stage IVA.

## Data Availability

The data presented in this study are not publicly available due to institutional restrictions but are available from the corresponding author upon reasonable request.

## Supplementary Materials

The following supporting information can be downloaded at: https://www.mdpi.com/article/10.3390/jcm15041552/s1, Figure S1. Overall survival (OS) according to first-line EGFR-TKI generation in the overall cohort; Table S1. Detailed classification of uncommon EGFR mutations; Table S2. Cox proportional hazards analysis for progression-free survival according to TP53 functional subtypes (corresponding to Figure 5).

## Abbreviations

NSCLC	non-small cell lung cancer
EGFR	epidermal growth factor receptor
TKI	tyrosine kinase inhibitor
EGFR-TKI	epidermal growth factor receptor tyrosine kinase inhibitor
PFS	progression-free survival
OS	overall survival
HR	hazard ratio
CI	confidence interval
DBD	DNA-binding domain
OD	oligomerization domain
WT	wild type
NGS	next-generation sequencing
1G/2G	first- or second-generation
3G	third-generation
