# Supplementary material for: TP53 Functional-Domain-Specific Mutations Define Distinct Clinical Outcomes in EGFR-Mutant Non-Small Cell Lung Cancer Treated with EGFR Tyrosine Kinase Inhibitors
Source: J Clin Med. 2026 Feb 15;15(4):1552. doi: 10.3390/jcm15041552 (PMC12941704; doi:10.3390/jcm15041552)
Supplement: Supplementary file 1 [file jcm-15-01552-s001.zip › jcm-4078632-supplementary.pdf]

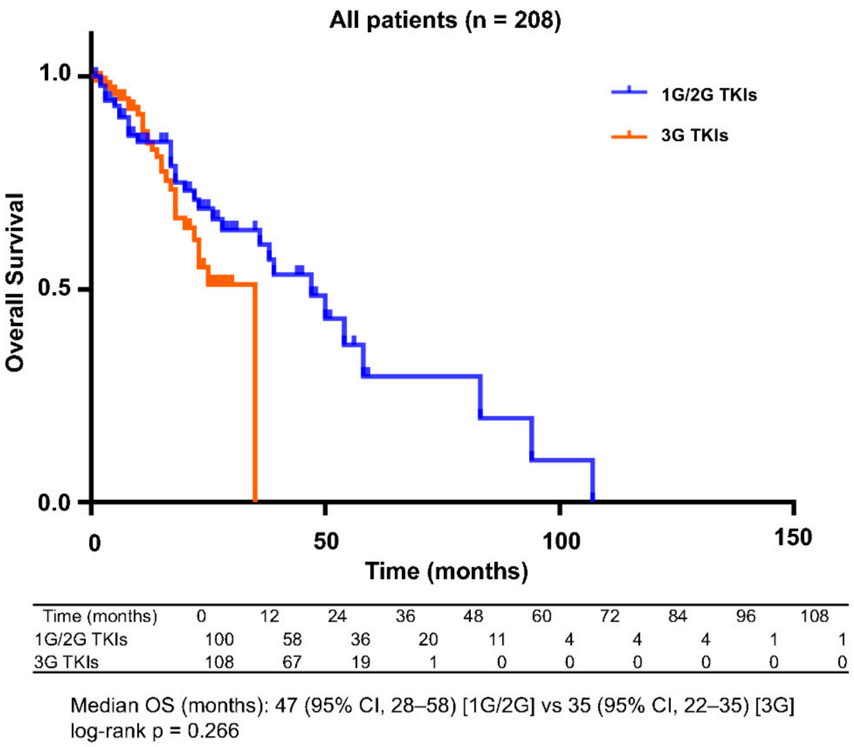

Figure S1. Overall survival (OS) according to first-line EGFR-TKI generation in the overall cohort. Kaplan–Meier curves for overall survival (OS) are shown for patients treated with first- or second-generation EGFR-TKIs (1G/2G TKIs; blue line) and those treated with third-generation EGFR-TKIs (3G TKIs; orange line) in the overall cohort (n = 208). Numbers at risk are shown below the plot.

Median OS was 47 months (95% CI, 28–58) in the 1G/2G TKI group and 35 months (95% CI, 22–35) in the 3G TKI group. No statistically significant difference in OS was observed between the two treatment groups (log-rank p = 0.266).

Table S1. Detailed classification of uncommon EGFR mutations.

Category	EGFR mutation pattern	n
Compound mutation involving L858R	L858R + T790M	8
	L858R + T790M + C797S	2
	L858R + G729A	1
	L858R + E709G	1
	L858R + L718Q	1
	L858R + S768I	1
	L858R + I759M	1
	L858R + L792H + T790M	1
Compound mutation involving exon 19 del.	Exon 19 deletion + T790M	6
	Exon 19 deletion + I706T	1
	Exon 19 deletion + V769M + G724S	1
Atypical exon 19 deletion variants	E746_A750del	6
	L747_S752del	1
	L747_P753delinsS	2
	E746_T751delinsV	2
	L747_A750delinsP	1
Exon 20 insertion / duplication	A763_Y764insFQEA	2
	D770_P772dup	1

	I740_K745dup	1
	A767_V769dup	1
Uncommon point mutations (single)	L861Q	2
	G719A	1
	G719S	2
	S768I	1
	G779F	1
Uncommon compound mutations (non-L858R)	G719C + S768I	1
	G719S + L861Q	1
Total		40

Hazard ratios estimated using Cox proportional hazards models corresponding to each panel are provided in **Table S2**.

Table S2. Cox proportional hazards analysis for progression-free survival according to TP53 functional subtypes (corresponding to Figure 5).

**Analysis group**	**TP53 subtype (vs WT)**	**HR**	**95% CI**	***p* value**
(a) All patients (n = 208)	Other mutations	1.33	0.75–2.38	0.335
	DBD-involved	1.73	1.13–2.65	0.012
(b) 1G/2G TKIs (n = 100)	Other mutations	1.56	0.69–3.54	0.283
	DBD-involved	2.93	1.62–5.31	<0.001
(c) Common EGFR + 1G/2G TKIs (n = 70)	Other mutations	1.27	0.43–3.75	0.661
	DBD-involved	2.42	1.13–5.17	0.022
(d) 3G TKIs (n = 108)	Other mutations	1.19	0.52–2.72	0.684
	DBD-involved	1.10	0.58–2.08	0.774

Cox proportional hazards models were fitted with TP53-WT as the reference group. PFS was defined as the time from initiation of EGFR-TKI therapy to disease progression or death.
